# Analyzing Russian Media Policy on Promoting Vaccination and Other COVID-19 Risk Mitigation Measures

**DOI:** 10.3389/fpubh.2022.839386

**Published:** 2022-04-29

**Authors:** Ivan Stepanov, Nadejda Komendantova

**Affiliations:** ^1^School of International Relations, St. Petersburg State University, St. Petersburg, Russia; ^2^Cooperation and Transformative Governance Research Group, Advancing Systems Analysis Program, International Institute for Applied Systems Analysis, Laxenburg, Austria

**Keywords:** COVID-19 discourse, media influence, information and communication strategies, coronavirus measures, public perceptions, linguo-cognitive analysis, Russia

## Abstract

The coronavirus disease 2019 (COVID-19) pandemic has resulted in many tangible and intangible losses. To manage the risk of the pandemic and to mitigate its further spread, governments of many countries applied various pandemic risk mitigation measures. Media campaigns played a particularly large role during the pandemic, too. In addition, social media grew in importance because of the spread of technologies and as a result of the increased attention to information about COVID-19. Media information strongly influenced both the public perception of COVID-19 risk and decision-making processes and choices, which people made regarding risk reduction measures during the pandemic. Moreover, media information has had a major impact on the effectiveness and efficiency of various countries' risk management actions. Therefore, the purpose of this article is to investigate the influence of the Russian media on the population's perception of risk, and to address the question about which linguistic and psychological methods they used to shape different media discourses about the COVID-19 pandemic. Thus, we analyzed media discourses as a part of the case study of COVID-19 risk management in the Russian Federation. The theoretical basis of the study includes mass communication theories. The methodological basis consists of linguo-cognitive analysis of empirical materials for specific political-philosophical, linguistic-publicistic, and sociopsychological functioning.

## Introduction

The coronavirus disease 2019 (COVID-19) pandemic has caused numerous tangible and intangible losses, as well as various negative cascading effects on national and global economies. To reduce the risk of the virus spreading, governments of different countries introduced various risk mitigation measures, which ranged from partial restriction of the economy to complete lockdown. The effects of these measures also varied in effectiveness and efficiency.

The media played an important role during this public health crisis and had a significant impact on people's behavior during the pandemic as well as on their adoption of risk reduction measures. In addition, the media gained more importance during this crisis because of the increased attention to various pieces of information about the virus, its spread and mitigation, and the availability of various theories about its origins and causes. The media landscape was characterized by the spread of information from both official and unofficial sources, as well as of various kinds of contradictory statements, rumors, misinformation, and even misleading news. Thus, the media has strongly influenced the perception of COVID-19 risk and led to various misconceptions, assumptions, and different assumptions.

The scientific novelty of the study lies in its topic, given the increased importance of social media, which has not been extensively addressed in previous studies. The volume of research findings on this topic is currently growing. It also consists of combining studies on media discourses and risk perception with further application of the analysis of news stories and social media messages for the specifics of their political-philosophical, linguistic-publicistic, and sociopsychological functioning. The research question of this article focused on different discourses and how these discourses shape risk perceptions and influence people's decision-making and choices during the COVID-19 pandemic. An empirical material was collected from a case study of the Russian Federation, which is relevant because of the rich media landscape characterized by different discourses and because of various measures, including its own developed vaccine, that were taken to manage the risk of COVID-19 and reduce the risk of the virus spreading.

The methodology of this study included various steps:

- Clarification of the theoretical assumptions, conceptual framework, and methodological basis of the study.
- Selection and systematization of text materials.
- Determination of the sociohistorical specificity of COVID-19 discourse in Russia.
- Consideration of media communication strategies.
- Analysis of the country's media sphere in terms of its influence on public awareness of COVID-19.

Our research had the following hypothesis*:* Russian COVID-19 media discourse is a structurally and instrumentally developed and widely represented phenomenon with a significant functional potential and is a linguistic and sociocultural resource. The significance of this study is determined by the fact that the materials of this study systematize and supplement the available information about the cognitive mechanisms of discourse interpretation and the functional potential of sociopsychological mechanisms. The materials of this study also determine the directions of future research, focused on the study of methods of complex linguo-cognitive interpretation of contemporary COVID-19 discourse.

## Theoretical Background

The relevance of the analyzed problem has predetermined its broad consideration in the modern scientific community. For example, when investigating media influence during the pandemic, the authors focus their attention mainly on studies Q25 on framing as one of the main information mechanisms. The emphasis of scientific studies is placed on reviewing and identifying the most popular frames in the global media environment (1), determining the influence of framing in the context of promoting certain risk mitigation measures both in the United States (2) and Russian information spaces (3). It is also worth mentioning several studies examining in detail the rumors, myths and hypotheses associated with COVID-19 and their direct connection to the perceptions of the pandemic. Thus, aspects considered range from climate and natural factors (4, 5) to the dis- and misinformation disseminated in social and mass media sources (6).

Therefore, the theoretical basis of our study was expanded and included several aspects of the intended analysis of COVID-19 media discourse in the Russian Federation. These aspects included political-philosophical, linguistic-publicistic, and sociopsychological aspects. This theoretical framework defined our theoretical-analytical study and the choice of categories for the subsequent analysis of the empirical material.

The concept of discourse plays an important role in our research and is defined as text consisting of communicative language units, sentences and their combinations into larger unities that are in a continuous semantic connection, which allows us to perceive it as an integral entity [(7), p. 8]. On this basis, more relevant to this study, the notion of COVID-19 discourse is defined as a set of speech products, recorded in writing or from memory, which represent meanings that define actions and events in the context of the COVID-19 pandemic [(8), p. 11].

We further identify the political-philosophical, linguistic-publicistic, and social-psychological aspects of this discourse.

The political-philosophical aspect examines the general attitude of the state to the role and functions of the media in the constructed national system. To this end, we turned to a review of the main normative theories of mass communication appropriate for this study:

- Authoritarian theory demonstrates the sociopolitical conditions for the media in which they can only be sustained if they take a loyal position and remain neutral *vis-à-vis* the government or are deliberately used as an instrument of repressive state power (9).
- Libertarian theory assumes that people are free to publish whatever they like but that they are responsible for all the consequences of their activities that violate human rights and the legitimate demands of society (9).
- Social responsibility theory presents the media as an independent and self-regulating resource with important functions in society (especially regarding the objective and pluralistic reflection and the promotion of democratic politics) and with certain obligations to it (9).
- Development media theory advocates media support for the existing regime and its efforts to ensure economic development, which helps society, thereby ignoring freedom of speech and of the press (10).
- Democratic-participant media theory is based on rejection of commercialization and monopolization of private media and centralization and bureaucratization of public broadcasting institutions, established in accordance with the norms of social responsibility, advocating diversity and horizontal communication links (10).

The linguistic-publicistic aspect is the most significant for this study. It consists of the analysis of thematic and lexical diversity in the context of agenda-setting and cognitive mechanisms of discourse interpretation (media influence) applied. This combination directly shapes the main direction of information policy within the framework of a general official or unofficial strategy.

The term “agenda-setting” refers to a set of media products that influence which issues, persons, and topics are perceived as the most important of the day (11). Thus, the media can write about some issues and ignore others by choosing the appropriate emphasis of description and argumentation.

In connection with the linguistic-publicistic aspect, one of the objectives of this study is to identify and analyze the main cognitive mechanisms of discourse interpretation, whose main purpose is to organize the processes of audience perception at an almost unconscious level, focusing its attention on the necessary information in the most appropriate place and time for this resource:

- Framing is used as a technique of forming and activating specific associations in the audience's memory to focus their attention on certain thematic features and thereby influence their subsequent behavior.
- Priming is based on a set of facts that are specifically selected and presented as a coherent image of an event that fits the perception of the media and the needs of the audience.
- Storytelling involves the presence of a certain narrative structure in the text, shaped in such a way that the events and facts described form a cause-and-effect relationship. This description leads the audience to conclusions that are convenient for the media source [(12), p. 218–235].

The sociopsychological aspect of the conducted research is in the specification of the linguistic-publicistic one, as it allows for revelation of certain properties of people's perceptions and cognitive models. Therefore, one of the tasks is to understand the influence of media discourse on COVID-19 risk perception and the level of awareness among people, as well as the forms of this media discourse, including the following:

- Informing is a type of information, devoid of manipulative techniques, about events and phenomena that an audience needs to know.
- Infecting is carried out by mass non-directed transmission of a mood that has a large emotional charge, the intensity of feelings and passions.
- Indoctrinating implies an active and personalized impact of the media source on the audience based on its emotional readiness to receive a certain attitude to action.
- Persuading is the formation of a certain system of attitudes and principles of personality based on both logical evidence (to a greater extent) and sense-value associations.
- Imitating is a sociopsychological mechanism of communication, which provides the reproduction of certain patterns of behavior by the audience, considering its experience and the circumstances of reproduction (13).

The considered theoretical basis allowed us to formulate the necessary categories for the subsequent analysis of the empirical material and organize the structure of the entire study.

## Methodology

During the study, we applied various research methods to understand media discourses and their impact on risk perception [the continuous sampling method, content analysis, critical discourse analysis (with the use of the MaxQGA software package), and statistical analysis].

Empirical data were obtained from various media messages and social media content. To identify media items for analysis, we used the continuous sampling method, which is a multistep approach to capture all occurrences of items of interest to the researcher. In the first step, we selected media messages according to a predetermined principle. Initially, from the variety of journalistic texts, we had to select examples relevant to the object of study chosen in this article, namely the Russian COVID-19 media discourse.

The period of publication of the material in the media was the main criterion for selection. We chose different time periods, beginning with the first report of the COVID-19 pandemic in the media in January 2020 and finishing with the most recent publications at the time of this study (July 2021). Then, it was decided to distinguish these stages statistically, i.e., according to the morbidity dynamics reported by the Federal Service for Surveillance of Consumer Rights Protection and Human Welfare (Rospotrebnadzor) (14). In this study, the empirical material was collected starting from the period with a steady increase in the number of incidents to the period with a steady decrease in the number of incidents (reaching its plateau).

Media popularity was the second criterion. We selected 110 media publications with the highest citation index, i.e., an index of citations between publications, tracing the impact of an article upon later ones (15). This included three media agencies, RIA, Interfax, and RBC (16). Furthermore, 20 articles for the period of the first constraints and 30 articles for each of the waves of coronavirus were selected. This material was selected gradually and analyzed concurrently, so that we could trace and capture all the encountered trends and patterns. By doing so, we managed to obtain and formulate reliable conclusions without addressing an excessive amount of the material. It was also important for us to collect the most popular articles among the audience to be able to more accurately track the effectiveness of the media methods used. Therefore, each item of the empirical material collected has more than 20,000 unique views.

We analyzed the content of the messages by content analysis, a research method used to describe the content of communication objectively, systematically, and quantitatively. The effectiveness of this method was shown in other studies analyzing great amounts of COVID-19 information materials for media framing as one of the main cognitive mechanisms of discourse interpretation (1). Thus, from the received volume of texts, we identified each element corresponding to the subject of our study, revealing the distribution of the selected material into predetermined thematic clusters (danger of COVID-19, coronavirus in Russia and the world, political decisions, introduced restrictive measures, vaccination, and public perception and behavior) considered as the most relevant topics for contemporary Russian COVID-19 discourse and, therefore, acting as semantic units of the research. Determining the presence of the above-mentioned clusters and considering possible differences in their distribution (and consequently their effectiveness), we applied the method of content analysis at two levels: for the abstracts and for the whole text. Thus, this method allowed us to calculate and characterize the main emphases of information sources (during each research period independently from each other), grouping them into categories suitable for further interpretation.

An important task of this study was to examine the strategies of informational influence of the media. Since sociopolitical practices, discourse, and public awareness on COVID-19 are in a dialectical relationship with other social dimensions and it seems necessary to emphasize this relationship, we turned to critical discourse analysis to characterize models of state-society interaction on COVID-19. In this stage, we also used the capabilities of the MaxQGA software package for the most effective textual research, which allowed us to compare and trace the relationship among features of the emerging COVID-19 discourse in Russia and the sociopolitical circumstances discussed above.

An analysis of public perception of COVID-19 risk was performed based on the available empirical data from 27 sociological surveys conducted among the Russian population during the time period that we selected for analysis. The main source of this empirical data is the non-governmental research organization Levada-Center. Here, we conducted statistical analysis to tackle this issue. On the first stage, this method was useful in adjustment and standardization of parameters of this study so that we could better identify and investigate deviations from the established norm. These parameters were formulated on the basis of the predetermined thematic clusters, encountered trends and patterns in the empirical material, arising hypotheses, and aspects of results interpretation. As mentioned above, the sociological material was also selected and analyzed gradually according to these factors. By doing so, in the second stage, we managed to obtain and formulate reliable conclusions without addressing an excessive amount of the material. As a result, we determined the change in correlation between the selected variables and the exact impact of these changes (where applicable): Russian media policy regarding public awareness and fear of COVID-19, trust in official information, the government and its risk mitigation measures, and public attitudes toward vaccination. The analysis of data from the content analysis, as well as from the Levada Center, allowed us to interpret and develop final conclusions about the influence of discourses on COVID-19 risk perceptions.

## Results

### Description of Case Study

Any analysis of COVID-19 media discourse is impossible without a detailed consideration of the sociohistorical conditions of its implementation. Therefore, we divided the media discourse into several periods when different risk mitigation measures were implemented. These periods are as follows:

#### The First Constraints (20 January−10 February)

On January 31, 2020, the first two cases of coronavirus infection were registered in Russia, but even before that, the federal and regional governments of the Russian Federation had already taken measures to prevent the entry of the coronavirus into the country and to contain its further spread. Thus, the first test systems to detect coronavirus were developed and put into production, passenger rail and air traffic was restricted, and a state of emergency was introduced in several border regions.

#### The First Wave of the Pandemic (17 March−21 June)

This period of the spread of coronavirus infection in Russia can be characterized as a very contradictory phase. On the one hand, the state provided material support to some groups of the population, medical personnel, and affected enterprises. Also, compared to several other countries, the Russian Federation managed to get through the first wave of the COVID-19 pandemic with relatively low rates of infection and mortality. On the other hand, this result was achieved, as in many other countries, thanks to unpopular social and economic policy decisions, such as the introduction of a new concept of “self-isolation”, which implied a 6-week period of unemployment (with employer-paid wages) and strict quarantine measures (permit system, administrative responsibility, mandatory use of masks and gloves, etc.). The implementation of such risk mitigation measures led to public protests in several regions.

#### The Second Wave of the Pandemic (7 November−15 January)

The fall of 2020 was marked by an increase in the incidence of COVID-19. At the same time, widespread vaccination against COVID-19 began in all regions of Russia, which, however, proceeded at a rather slow pace compared to other countries (about 5%) (17).

#### The Third Wave of the Pandemic (5 June−23 July)

In June 2021, there was a new spike in the incidence of COVID-19 (also due to the spread of the Indian and British strains). In response, the government proposed several new measures to contain and overcome the coronavirus. First, to speed up vaccination rates in some regions, compulsory vaccination for several professions was introduced. This contradicted previous statements by politicians about the voluntary nature of vaccination. At the same time, the population was confused by the lack of alternatives to Sputnik V (foreign vaccines were not registered, CoviVac quickly ran out, and the effectiveness of EpiVacCorona was not confirmed). Second, a new pass system was introduced. This system did not allow people to visit public places without a QR-code. The introduction of this system caused several public protests.

The results of our analysis allowed for making the following conclusions about various periods of reporting about the COVID-19 pandemic.

### The First Constraints

In the second half of January and first days of February 2020, particular attention was paid to the mechanisms of media influence and behavioral economics techniques to raise awareness of the COVID-19 risk and influence perceptions of the virus and risk reduction measures.

During this period, the focus of COVID-19 discourse in Russia was on the situation in China and the world (including evacuation of Russian citizens). Thus, Russian media resources were able to take advantage of this external unfavorable epidemiological situation and, on its basis and without the need to violate the principle of truthfulness of the illustrated picture, to build certain aspects of their own information strategy. Within this agenda, the media sources were aimed at the increasing danger and contagiousness of the new infection. This was realized through frequent references (in about 80% of the articles) to statistics on the number of cases and deaths in China and in many other countries where cases of infection have been registered.

The use of indoctrinating (60%) in combination with priming (44%) while covering coronavirus-related events led to increased awareness of the importance and relevance of the COVID-19 pandemic topic:

- *Scientists from an Australian research institute have successfully grown a new coronavirus*. < …>*Already 132 people have fallen victim to a new coronavirus in China, and almost 6,000 have been infected* (in this example, there is no need to publish negative statistics in an article covering another event; this demonstrates the aim of an information source to create and reinforce the image of the coronavirus danger).

Data from a Levada-Center poll of the Russian population showed that in January only 15% of respondents thought that the topic of COVID-19 was important. However, in February 2020, this number rose to 40% (18). Only 1% of all respondents said they knew nothing about the COVID-19 pandemic (19). Media campaign also influenced people's perceptions of the seriousness of concerns about the COVID-19 pandemic. Initially, most people noted that they had little concern about the COVID-19 pandemic and hoped that the virus would remain localized and would not spread across the country. However, in February 2020, the number of people concerned about the virus rose to 30%. In March 2020, the number of those concerned rose to 44% (19, 20).

It is also important to consider other topics and events that are potentially more controversial and important for the audience and, therefore, have a possibility to be displaced from the central information focus. It should be noted that the selection of these topics was decided to be made in this stage as well as at the stage of the first wave of the pandemic. This approach makes it possible to analyze both the actual goals of media activity at the time and the information base being formed for planned ambiguous events and decisions. Thus, such topics include, first, proposals to amend the Constitution of the Russian Federation (January 15) and to nullify previous presidential terms (March 10, 2020).

If we dwell on each of these topics in more detail, it is worth noting that the message about the amendments to the Constitution, to a lesser extent, implies the implementation of a parallel information campaign aimed at redirecting the audience attention. Thus, this is primarily because the population is highly aware of the proposed initiative (84% of respondents, with 13% considering this event to be the most important during the period under review); it has incomplete understanding (68%) but high approval of the amendments (over 50% for each individual item) (21–23).

On the other hand, the proposal to nullify the previous presidential terms was met with much skepticism (40% of respondents supported this initiative, while 34% were against it; it is also important that only 24% of the respondents were going to attend a referendum on the adoption of these amendments, which, in general, made the legitimacy of the project more difficult) (24). Therefore, it is interesting that despite its ambiguity and importance, this topic was little reflected in the media focus and became the most memorable event for only 3% of respondents (“coronavirus pandemic” 67%, “discussion of the amendments” 13%) (23).

Another media topic was the restrictions imposed in Russia to prevent the entry and spread of the coronavirus in the country. It is also worth mentioning such important issues for the media environment of this period as difficulties in the healthcare system (related both to shortages of certain goods and the first cases of coronavirus infection in Russia). On this basis and with the use of persuading (40%) and framing (56%), the media sources aimed at creating an atmosphere of confidence and offering a solution to the problem; they were forming:

- *Both infected persons are Chinese. They are now under treatment and are completely isolated* (in this example, an information source emphasizes nationality (meaning imported but not spreading infection) and the measures taken on the cases described).

Moreover, this strategy was meant to increase the level of public trust in the government, which, as stated above, was crucial to this period. Thus, this activity stands out against the general background of the danger of coronavirus and is contrasted with other countries (especially China, Italy, and the United States). For this purpose, information sources aimed at extensive coverage of risk mitigation measures introduced by Russia, as well as controlled situation with cases of COVID-19 in the country and the evacuation of citizens from abroad (also underlining the humanitarian aid offered to affected states).

As mentioned earlier, in February 2020, the number of people concerned about the virus rose to 30%, but 17% still rated the risk of COVID-19 as a low level of serious danger (19, 20). All of this was also reflected in the population's trust in disaster risk reduction authorities and their ability to control the risk of COVID-19 (25). The level of trust increased because of media campaigns about successes in Russia to control the spread of the COVID19 virus. Moreover, the high level of trust also corresponded to a high need for security, especially during periods of crisis (26).

The media campaign of the period of the first constraints achieved the desired results. However, there were some major problems caused by this activity that could be avoided. Thus, despite the media reports during this period being characterized by both positive and negative sentiments (47 and 53% respectively), the main focus was placed on the increasing danger and contagiousness of the new infection. On the one hand, because an emphasis was put on the threat to the population, this issue managed to become quickly and firmly embedded in the minds of the audience. On the other hand, this was overflowed and led to the major increase in people's psychological tension, i.e., a feeling of psychological strain accompanied by discomfort, uneasiness, and pressure (27, 28). Therefore, media activity should have monitored the changing audience perceptions and attitudes, and, by reaching the most appropriate level of its awareness, shifted the concentration onto the second aspect, which is on creating an atmosphere of confidence, as it suggests more diverse and less destructive methods of influence.

### The First Wave of the Pandemic

The second phase of the COVID-19 discourse we analyzed includes the period from the second half of March to June. These months saw a significant increase in COVID-19 cases; in addition, a number of risk mitigation measures, ambiguously perceived by society, were introduced.

It is appropriate to analyze and compare the peculiarities of this information campaign, considering the conditions of its implementation, which have been discussed earlier. In March, prior to the period analyzed, 44% of respondents feared contracting coronavirus, 47% thought the healthcare system was ready for the pandemic, and only 24% had little trust in official information (20, 29, 30). Based on this, as well as previous experiences with COVID-19 discourse, media sources continued their own information strategies; and the presented situation can be again characterized as dangerous but contained and controlled by the government. Thus, the main reassuring topics of media discourse during this time were internal decision-making processes, successes, and failures in the fight against the pandemic, and discussion of containment measures and their implementation, mainly in Moscow but also in other regions, such as self-isolation and the attitude of various publicly known personalities toward it.

Regarding the phase of the first constraints, the analysis of the mechanisms of media influence and psychological techniques, among which the quantitative representation differs, again comes to the fore. Thus, the media framing (77%) of that time shows the correctness of risk reduction actions and existing problems, such as the need for and effectiveness of restrictions, which are justified, for example, by an argument to avoid the scenarios of other countries:

- *The rate of coronavirus incidence in Russia has been reduced significantly, said the head of the Federal Medical and Biological Agency, Veronika Skvortsova. “In fact, we have already been at a plateau in the number of new cases for the last week”* (framing also implies using “masking” terms, for example, “plateau”).

Media mechanisms and techniques such as framing, priming (22%), and indoctrinating (56%) were also competently used in discussing the increase in the number of infected and deceased people:

- *In early March, WHO declared a pandemic outbreak of coronavirus infection spreading worldwide from China. According to the organization*'*s latest figures, about 750,000 people have already been infected and more than 36,000 have died. The total number of COVID-19 patients in Russia has reached 2,337 (1,613 in Moscow), and 121 of them have been cured*.

Thus, according to the media, the low mortality rate is explained by the fact that healthcare is organized systematically and patients with COVID-19 can receive the necessary treatment. When describing a fatal case, the emphasis is placed on the fact that the patient had comorbidities or was an elderly person. The high morbidity rate is also due to the increased frequency of testing for coronavirus and the reluctance of the population to comply with self-isolation. In contrast to other countries, not only statistics on the number of cases and deaths are reported but also the number of people cured. In arguing the need for quarantine, mortality figures are also provided.

Compared to the period of the first constraints, other psychological techniques, such as imitating (17%), were used for the first time in this period. The following case is an example of it: the head of the government and the chief doctor of the infectious diseases hospital decided to observe self-isolation, and most citizens decided not to violate anti-quarantine restrictions during the May holidays:

- *Most Russians are not planning to violate their self-isolation regime during the May holidays, but to spend time at home or in the countryside* (this example emphasizes a mass positive example of appropriate social behavior).

The results of these strategy influenced people's reactions; however, the desire to legitimize the measures taken by government proved highly ambiguous. Thus, Levada-Center results show that the media campaign was successful in demonstrating the need for risk mitigation measures. In April, 48% of respondents fully approved the COVID-19 containment solutions. In May 2020, the figure was already 66%. This increase demonstrates the success of the information policy (31, 32). Surprisingly, however, the government's rating was 10 points below the pre-pandemic levels (from 69% in February to 59% in May 2020) (33). This can be explained by a combination of reasons.

First, media activities were designed to again emphasize the dangers of the coronavirus. On the one hand, this media campaign was successful in raising awareness of the coronavirus risk. In April 2020, already 57% of respondents said they were afraid of coronavirus infection. Interestingly, the so-called saturation limit was reached shortly thereafter. After this limit, there were no noticeable fluctuations in the increase in the number of people fearing coronavirus (30).

On the other hand, as mentioned above, because of the emphasis on the threat to the population, its overabundance and saturation limit and the high level of public trust in the official information led to the major increase in people's psychological tension. Thus, from January to May 2020, a stable growth was demonstrated by sales of sedatives (+40%), and people have four times more often turned to psychologists compared to the same period of 2019 (27). This negative condition has also increased as a result of the global economic downturn and loneliness as a consequence of reduced social contacts.

This problem has a significant negative impact both on society as a whole and on its political and other components, in particular. For example, depression, as one of the forms of mental disorder, has become one of the main causes of decline in people's ability to work: labor productivity is reduced four-fold, the number of all kinds of accidents increases because of reduced concentration, and the number of sick leaves related to psychological problems reaches 30–50% of the total. As a result, this also worsens the negative factors described above (34). All of this was also reflected in the economic component: for example, as a result of the rush in demand for food and basic necessities, there was a shortage of them. It is interesting to note that in media sources this was, however, framed with the unscrupulousness of some producers exporting scarce products needed in the country.

Social tension developed on this basis (defined as a negative emotional state in society caused by pressure from the natural or social environment) was the third reason for the decreased level of political trust during this period. Critical points in the expression of this tension and consistent protest activity were political [noted in the period of first restrictions, the proposal to nullify the previous presidential terms and amend the Constitution (35)], economic [the crisis drop of GDP by 12 points (36)], and other factors [such as restrictive measures, disapproved by 32% of respondents (32)].

The first and second phases of the COVID-19 discourse demonstrated the unpreparedness of the media leadership to adapt to the new crisis conditions. Despite achieving the planned goals (i.e., raising the level of COVID-19 awareness and the level of public trust in the risk mitigation measures taken), the media failed to minimize the negative impact of the implemented information policy. As noted earlier, the key missing tool was the monitoring of public perception.

### The Second Wave of the Pandemic

The peak of the second wave of the pandemic in Russia came in November 2020–January 2021. The focus of media attention was naturally on the rise of COVID-19 and the beginning of large-scale vaccination, with a significant informational role given to Moscow as the leader in political decision-making and the anti-COVID campaign.

The main distinguishing feature of the discourse on COVID-19 during this period was a particularly large amount of indoctrinating (80%) and an overall negative background of the information presented (64%):

- *Protsenko previously reported that a third of patients with COVID-19 die within the first 72 hours of hospitalization. He explained that this is most often due to hypoxia and thromboembolism. Meanwhile, those patients who died later, died in most cases from septic complications. The bacteria that lead to death from these kinds of complications develop rapidly in the infected body* (in this example, indoctrinating is realized through a detailed description of the negative implications of the disease in a publication that does not require such redundant information).

Elements of the discourse elicited a corresponding reaction from the audience. In this case, a parallel can be drawn with the periods of the first constraints and the first wave of the pandemic. Thus, information sources were again aimed at reinforcing the danger of the coronavirus through frequent references to statistics on the number of cases and deaths in Russia and the world. As noted earlier, the number of those who fear getting infected reached 57% in April, and then declined in the following months but peaked again to 64% in October 2020 (37). There are three possible reasons for this fluctuation. First, the saturation limit mentioned above may have been reached as a result of an overabundance of information about COVID-19 in the media. Second, there were relatively few cases of coronavirus during these summer months, which may have had a relieving effect. Third, and more interestingly, news of the first vaccine registration (August 2020) was perceived by a preponderance of distrustful, doubtful, and fearful people (38), which may have had a similar effect on the image of the COVID-19 pandemic.

It was the first Russian vaccine that was offered to the audience as a solution to the looming problem, representing the reliability of the Russian healthcare system and the controllability of the situation. To this end, a special role is played by constraints, which, while continuing to be described as necessary, gradually reveal a shift in functional emphasis toward a negative alternative to the vaccination process being promoted:

- *Earlier, the head of the city assessed the system developed in China, according to which entire neighborhoods were isolated within epidemiological measures taken against COVID-19. According to Sobyanin, it is completely inapplicable to Russia, although it is an “effective method” of combating the spread of coronavirus* (in the context of promoting vaccination, framing is used to describe the effectiveness and potential application of stricter quarantine measures).

This information strategy was to convince audiences, tired of continuing and increasing restrictions, that vaccination is the best choice for them. However, this approach had little effect on the overall vaccination rate, since, first, most people agreed with the introduction of and compliance with COVID-19 restrictions. Thus, compared to May 2020, the percentage of people approving the COVID-19 risk mitigation measures did not change significantly in October 2020 (with most respondents supporting both their introduction and repeal) (39, 40). Second, distrust in the vaccine offered was a greater factor in the population's decision to get vaccinated (38, 41).

Here of some interest is a survey that does not fall within our analyzed time period. According to the experts who took part in it, low vaccination rates among the population (about 5% at the time of the survey) are primarily due to anti-vaccination attitudes of the general population, lack of trust in existing Russian vaccines, and lack of awareness about the importance of vaccination (42). This poll partially contradicts other data in the point about the anti-vaccine stance, and in the fact that the distrust of the population persisted despite the appearance in the media of information about Sputnik V trials (38).

In this context, the survey on the reliability of official information about the number of people infected with coronavirus is also interesting. While in March only 24% of respondents thought this information was unreliable, in October 2020 it was already 61% (39). We have no evidence on whether this had a negative effect on the adoption of the anti-COVID campaign. However, this reveals a prospective correlation “trust in the vaccine, trust in the government, COVID-19 fear (trust in official information)”.

Compared to the previous phases, during the second wave of the pandemic, although an updated but proven strategy was used to legitimize government decisions against the background of an increased coronavirus danger, the main goals of information policy (i.e., promotion of the domestic vaccine) were not achieved. Again, we can observe that the focus of the media was placed on the increasing danger and hardness of the situation, even when attempting to create an atmosphere of confidence [for example, offering the choice between two unpopular and unfavorable measures (vaccination and restrictions)]. As a result, this period also saw an increased number of complaints of anxiety, stress, and other forms of psychological distress (43). The need to tackle this problem was also noted at the Russian legislative level (36).

Therefore, it was considered as promising to use more positive methods of influence. For example, the media should include information about the number of vaccinated (in Russia and the world) in combination with statistics about the number of cured, which will strengthen the positive association “vaccination = cure” and attract the attention of the audience to the mass positive example of behavior (the psychological mechanism of imitating). Of particular importance is also the frequent mention of information about the final stages of vaccine trials, which, according to the analysis of social surveys, many people lack to make a positive decision about vaccination (on the other hand, the publication of such results, for example, for the Sputnik V vaccine turned to be mostly unnoticed).

### The Third Wave of the Pandemic

At the time of this study, the third wave of the pandemic was underway. This influenced the selection of the empirical material in the limited period of June-July 2021. As in the previous period, the focus of information sources was on the increasing number of COVID-19 cases and promotion of the need for vaccination. A particularly large number of media reports focused on the situation in Moscow.

The discourse of this period, in comparison with the periods that we described earlier, is characterized by the presence of both common and distinctive features. Common is the use of psychological indoctrinating (68%) and the method of framing (73%). They are devoted, among other things, to accentuating the danger of COVID-19 and forming an image of a difficult but controllable situation:

- “*In any case, if you fall ill and have symptoms of a respiratory infection, gastrointestinal distress, high body temperature, you should stay home and get tested for SARS-CoV2. And to prevent this from happening, get actively vaccinated and continue taking precautions,” Pshenichnaya stressed* [in this example, an information source forms the favorable image of risk mitigation measures, emphasizing the negative implications common for a number of diseases and underlining the effectiveness of vaccination against them (even without mentioning COVID-19)].

However, in this direction, there is a noticeable deterioration in audience perceptions (roughly persisting since January 2021): thus, 43% of respondents are afraid of contracting the coronavirus, while 55% are not afraid (44). As in the previous period, there are several possible reasons for this fluctuation: overabundance of information about COVID-19 in the media and relatively few cases of coronavirus during previous months. Here we do not consider the factor of distrust in vaccination, as despite that most respondents were still not ready to get vaccinated, and this figure dropped by 7 points to 55% from its peak (19).

Also, because of this low level of trust and vaccination rate of the population, special attention is paid to the promotion of this aspect. Thus, as noted earlier, to speed up vaccination rates, compulsory vaccination for several professions as well as a new QR-code pass system were introduced. The possibility of avoiding these new restrictive measures and the disease itself is directly related to the readiness of the population to be vaccinated:

- *She added that social monitoring systems and QR-codes, and an order for mandatory vaccination against COVID-19 for 60% of workers in several industries had been introduced because of a desire to avoid strict quarantine*.

In this context, it is interesting to note the extremely rare mention of statistics on illnesses and deaths, which correlates with the interpretation of the data we obtained in the previous stages.

Since vaccination represents the greatest importance in the formed information agenda, it is reasonable to consider the results of surveys in this direction. Thus, as of July 2021, 43% of respondents were going to or had already been vaccinated against the coronavirus; 59% of respondents had been vaccinated to reduce the risk of severe disease; 15% of respondents were vaccinated because of work orders, and 14% were to have full access to all facilities (45, 46). These statistics demonstrate the prevalence of a competently constructed information campaign over artificially created difficulties resulting from the introduction of harsh restrictive measures. In this context, it is also important to note that these measures had an extremely negative impact on the perception of government decisions, whose approval rate dropped to a low of 40% during the pandemic (July 2021) (47).

At the same time, most respondents were not ready to get vaccinated (55%). This figure correlates directly with the prevalence of fear of getting infected (20% difference) and the level of approval of government activities (25% difference), which most likely indicates an individual's commitment either to the triangle “COVID-19 fear (trust in official information), trust in government, trust in the vaccine” or vice versa. It is important to note that the main reason for refusing vaccination was the fear of side effects and the lack of final test results (19).

This is directly related to another incompetent activity of media sources. Thus, the effectiveness and demand for Russian vaccines are demonstrated in contrast to their foreign counterparts. This should especially be avoided, as such “Russia vs. West” discourses have a negative impact on the goal of communicating the need for vaccination. Such discourses continue the narrative of contrasting Russian medicine with its foreign counterparts and lead to additional prejudices and doubts. On the contrary, the complex favorable and accepted image of vaccination should be created, based on which the advantages, importance, and safety of domestic vaccines should be subsequently explained.

## Discussion

Our results allow for us to develop recommendations for media strategy in terms of the considered political-philosophical, linguistic-publicistic, and sociopsychological aspects.

### The Political-Philosophical Aspect

The Russian information system, in general and in the case of the COVID-19 pandemic, is characterized by the role of the state, which aims to achieve certain economic, political, and social goals and objectives. Such a system makes it possible to achieve planned indicators, which makes the overall information strategy appropriate, especially during catastrophes and crises. Our results show that such a strategy creates a calming and reassuring atmosphere with a proposed solution in the form of the promotion of accepted political decisions (compliance with restrictive measures, vaccination, etc.). On the other hand, the study also demonstrates the need for additional monitoring and control of:

- the index of audience perceptivity and sentiment, which directly affects the state of the public (for example, psychological and social tensions resulting also in economic and political volatility), and
- the index of audience awareness, expressed in attaining a certain level of trust in official information. In this aspect, we also consider it expedient to form the information base not only for the planned but also for the expected actions and events in advance, which helps to prepare the audience for the perception of the expectedly ambiguous information. For example, within a certain period prior to the news about the creation of the first Russian vaccine, information sources should have reported the successes of the health sector, the effectiveness of vaccination in preventing other diseases, etc.

The results also show that discourses against dependency, foreign influence, or the formation of an opposition “Russia-West” should be avoided. Such discourses have a negative impact on the goal of communicating the need for vaccination. Such discourses continue the narrative of contrasting Russian medicine with its foreign counterparts and lead to additional prejudices and doubts. On the contrary, the narrative should be followed to create an image of the benefits, importance, and safety of both domestic and foreign vaccines.

### The Linguistic-Publicistic Aspect

Particular attention in this aspect is paid to the mechanisms of media influence. The analysis of these mechanisms has shown the effectiveness in achieving certain goals in the analyzed periods, which determines the legitimacy of its further use. Thus, within the framework of the above-mentioned strategy, framing (63%) plays a decisive role in presenting contradictory information in favor of the correctness of the state policy, as well as in refuting the criticism of foreign representatives. Priming (36%), on the other hand, acts more as a method of shaping and maintaining the image of the spreading and extremely dangerous COVID-19 pandemic and promoting domestic vaccines.

On the other hand, the analysis of the empirical material, however, revealed very few examples of storytelling (1%) (which is also relevant to few cases of imitating applied). This demonstrates the emphasis in information activities on selecting and describing a material about specific non-personalized events and actions. However, perhaps more attention should be paid to consideration and demonstration of human subjective feelings, experiences, and stories, which would make the broadcasted material more lively, understandable, and close to the audience. For example, this method is promising for application with several particularly popular topics including: messages about famous personalities, information about possible future (consequences of coronavirus, and encouraging stories from the first vaccinated people (as an option)).

It is also important to consider the frequent mention by information sources of statistics about the number of cases and deaths, as well as numerous countries in which cases of infection have been reported. Since this technique helped achieve the planned results, we consider it promising to include information about the number of vaccinated (in Russia and worldwide) in combination with statistics about the number of cured, which will strengthen the positive association “vaccination = cure” and attract the attention of the audience to the mass positive example of behavior (the psychological mechanism of imitating). Of particular importance is also the frequent mention of information about the final stages of vaccine trials, which, according to the analysis of opinion polls, is not enough for many people to make a positive decision about vaccination (despite the longstanding but unnoticed publication of these results, for example, for the Sputnik V vaccine). On the other hand, promotion of Russian vaccines should be moderate, not reaching the saturation limit and, consequently, resistance of the audience to the information offered.

Another component of the linguistic-publicistic aspect of information activity is the agenda being formed, in which the main topics were, of course, the growing number of infections and measures being taken to contain the COVID-19 pandemic (restrictive measures, vaccination), including focus on the situation in Moscow. It is also worth mentioning other popular topics that can be effectively used in further information campaign: forecasts about present and possible future pandemics, and possibilities to avoid or easily overcome a disease (with the help of traditional medicine, etc.).

### The Socio-Psychological Aspect

In this aspect, we have analyzed the socio-psychological mechanisms used by the media sources to influence certain characteristics of the audience's psyche and, thus, to popularize certain social actions. The greatest number of examples in the selected practical material includes indoctrinating (66%), often used in combination with priming (publication of statistical data), including for forming an image of a dangerous epidemiological situation and explaining the need for vaccination. Interestingly, the role of indoctrinating can be seen in how, after ignoring the entire group of -psychological mechanisms after the development of the first vaccines and turning to them during the second and third waves of the pandemic, there was an increase in the population's approval of the vaccination.

The next most popular mechanisms were persuading (23%) and imitating (11%), whose function was to increase acceptance of restrictive measures and vaccination. In contrast to indoctrinating, these methods were not used during all of the periods analyzed, which may be due to a lack of media confidence in their effectiveness. On the contrary, as noted above, imitating can be used to draw the audience's attention to a mass positive example of vaccination behavior (with the publication of statistical data) in a new but proven way. An important addition to the previous recommendation could be the publication of scientifically proven facts and opinions of experts and medical professionals (competent in infectious diseases), also indicating the number of vaccinated (persuading, imitating). All this can significantly increase public awareness of the importance and necessity of vaccination (and gradually more relevant revaccination).

**Figure 1:**
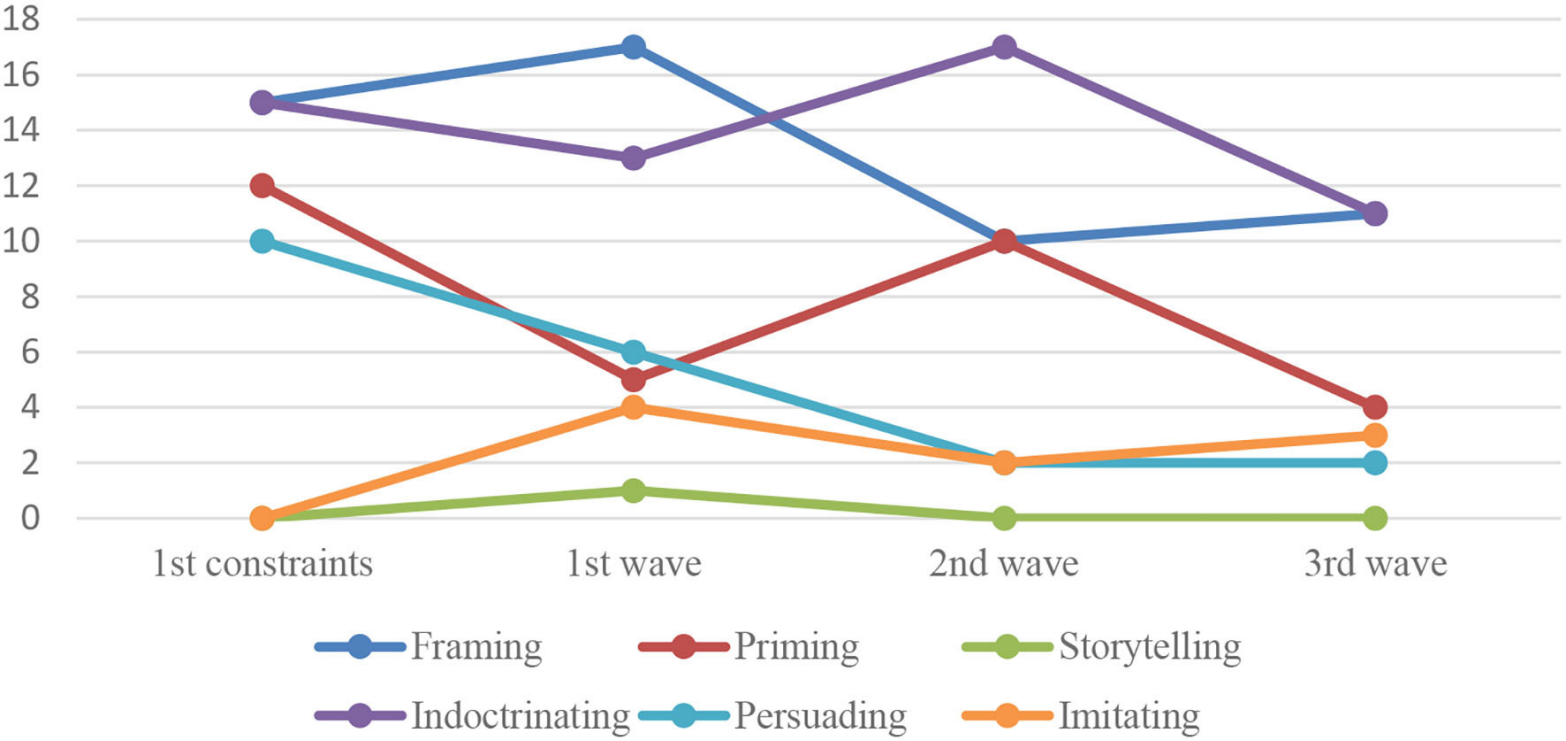
Distribution of media mechanisms over the study periods (number of cases).

**Figure 2:**
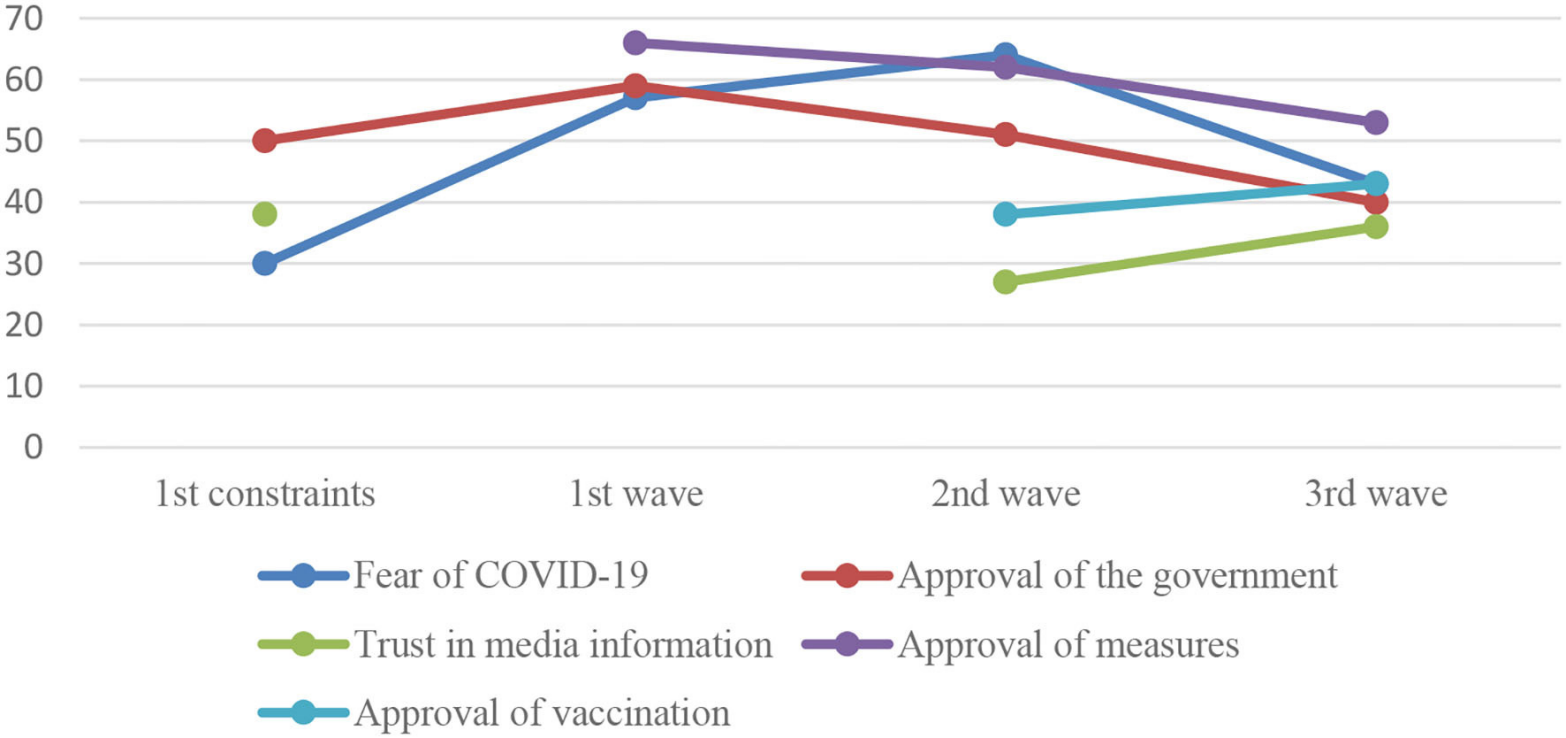
Fluctuation of public perception over the study periods (%).

Therefore, this article presents a multicomponent study on contemporary Russian COVID-19 media discourse, which includes both study on and generalization of traditional literature on the problem of mass communication and independent analysis of actual empirical material for the specifics of its political-philosophical, linguistic-publicistic, and sociopsychological functioning. Detailed statistics are presented in Figures 1, 2.

## Data Availability Statement

The original contributions presented in the study are included in the article/supplementary material, further inquiries can be directed to the corresponding author.

## Author Contributions

Both authors listed have made a substantial, direct, and intellectual contribution to the study and approved it for publication.

## Funding

The research was conducted with financial support from the Petr Aven Fellowship and from International Institute of Applied Systems Analysis in frames of the CORE Project (Agreement No. 101021746).

## Conflict of Interest

The authors declare that the research was conducted in the absence of any commercial or financial relationships that could be construed as a potential conflict of interest.

## Publisher's Note

All claims expressed in this article are solely those of the authors and do not necessarily represent those of their affiliated organizations, or those of the publisher, the editors and the reviewers. Any product that may be evaluated in this article, or claim that may be made by its manufacturer, is not guaranteed or endorsed by the publisher.
